# Sympathetic and Vagal Responses Elicited by Acute Stress in Rats

**DOI:** 10.7759/cureus.11602

**Published:** 2020-11-20

**Authors:** Eleni-Taxiarchia Mouchtouri, Panagiotis Lekkas, Foteini Delis, Emmanouil Pantelakis, Iordanis Mourouzis, Constantinos Pantos, Theofilos M Kolettis

**Affiliations:** 1 Cardiology, University of Ioannina, Ioannina, GRC; 2 Cardiology, Cardiovascular Research Institute, Ioannina, GRC; 3 Physiology, University of Ioannina, Ioannina, GRC; 4 Pharmacology, University of Ioannina, Ioannina, GRC; 5 Pharmacology, National and Kapodistrian University of Athens, Athens, GRC

**Keywords:** acute stress, sympathetic response, vagal response, heart rate variability, recovery

## Abstract

Introduction

Acute emotional stress triggers autonomic responses that affect sympathovagal balance. However, the temporal pattern of changes in each autonomic arm during stress and recovery remains unclear. Therefore, we analyzed separately sympathetic and vagal activity, elicited by acute unpredictable stress in a rat model.

Methods

Continuous electrocardiographic recording was performed during (32 minutes) and after (two hours) successive use of restraint and air-jet stress in 10 rats, whereas five rats served as controls. Sympathetic and vagal indices were calculated non-invasively after heart rate variability analysis. Voluntary motion was quantified during recovery, as an index of continuing anxiety.

Results

The sympathetic nervous system index increased during stress and remained elevated during the initial stage of recovery. The parasympathetic nervous system index decreased immediately after the onset of stress and remained low throughout the observational period. During recovery, voluntary activity was more pronounced in the stress group than in the controls.

Conclusion

Successive restraint and air-jet stress in rats increased sympathetic activity and decreased vagal activity. These changes displayed only partial recovery post-stress and were accompanied by enhanced voluntary motion. Our findings may be important in the evaluation of the cardiac electrophysiologic implications of autonomic changes elicited by acute emotional stress.

## Introduction

Acute aversive stimuli activate the endocrine, nervous, and immune systems that aim to maintain homeostasis. However, a rare, albeit potentially serious, untoward effect of the response to acute emotional stress is the onset of ventricular tachyarrhythmias [1]. Autonomic activation has been long implicated as the main mediator of such rhythm disturbances, via altering the electrophysiologic properties of the ventricular myocardium [2]. Based on its vast clinical importance, animal models of acute unpredictable stress are widely utilized, aiding the investigation of various aspects of the underlying physiology and pathophysiology.

Restraint [3] and air-jet stress [4] in rats have emerged as simple and effective translational models of acute emotional stress. These methods, alone or in combination, are in use for decades, and their effect on sinus heart rate (HR) has been described in detail. However, HR reflects overall sympathovagal balance, whereas the alterations occurring in each arm of the autonomic nervous system have not been adequately addressed; such assessment is important, given their distinct effects on cardiac electrophysiology [5].

In the present study, we examined sympathetic and vagal responses during acute unpredictable stress in rats, elicited by the successive use of restraint and air-jet stress. During an extended observational period post-stress, we also monitored voluntary activity, as an index of continuing anxiety [6]. Autonomic responses during and after stress were assessed by means of heart rate variability (HRV), derived from continuous electrocardiographic (ECG) recordings.

## Materials and methods

Animal study population

The experiments were conducted on 15 Wistar rats, on which stress was induced in 10 (443 ± 8 g), whereas five rats (452 ± 9 g) served as controls. We kept our animal population homogenous by including only male rats, given the previously reported gender-related differences in HR responses to stress [7]. The animals received humane care and had free access to food and water; they were housed in singles in standard plexiglas cages during the entire experimental period. Optimal environmental conditions were maintained in the facilities, in terms of temperature (20℃-22℃), humidity (70%), and light/dark cycles (12/12 hours). All procedures described herein comply with the Animal Research: Reporting of In Vivo Experiments (ARRIVE) guidelines and have been carried out in accordance with European legislation (2010/63/EU). The study protocol was approved by the institutional and regulatory authorities (Regional Municipality of Attika, approval number: 574219).

Telemetry

ECG was recorded telemetrically via miniature transmitters, implanted a minimum of five days prior to the induction of stress. Following intubation, the rats were ventilated (Model 7025, Ugo Basile, Verona, Italy) and anesthetized with 2.5% isoflurane (Abbott Laboratories, Abbott Park, IL, USA). The devices (TCA-F40, *Transoma*, New Brighton, MN, USA) were implanted in the abdominal cavity, with both leads sutured to the surrounding tissues to avoid motion artifacts.

Stress protocol

**Figure 1:**
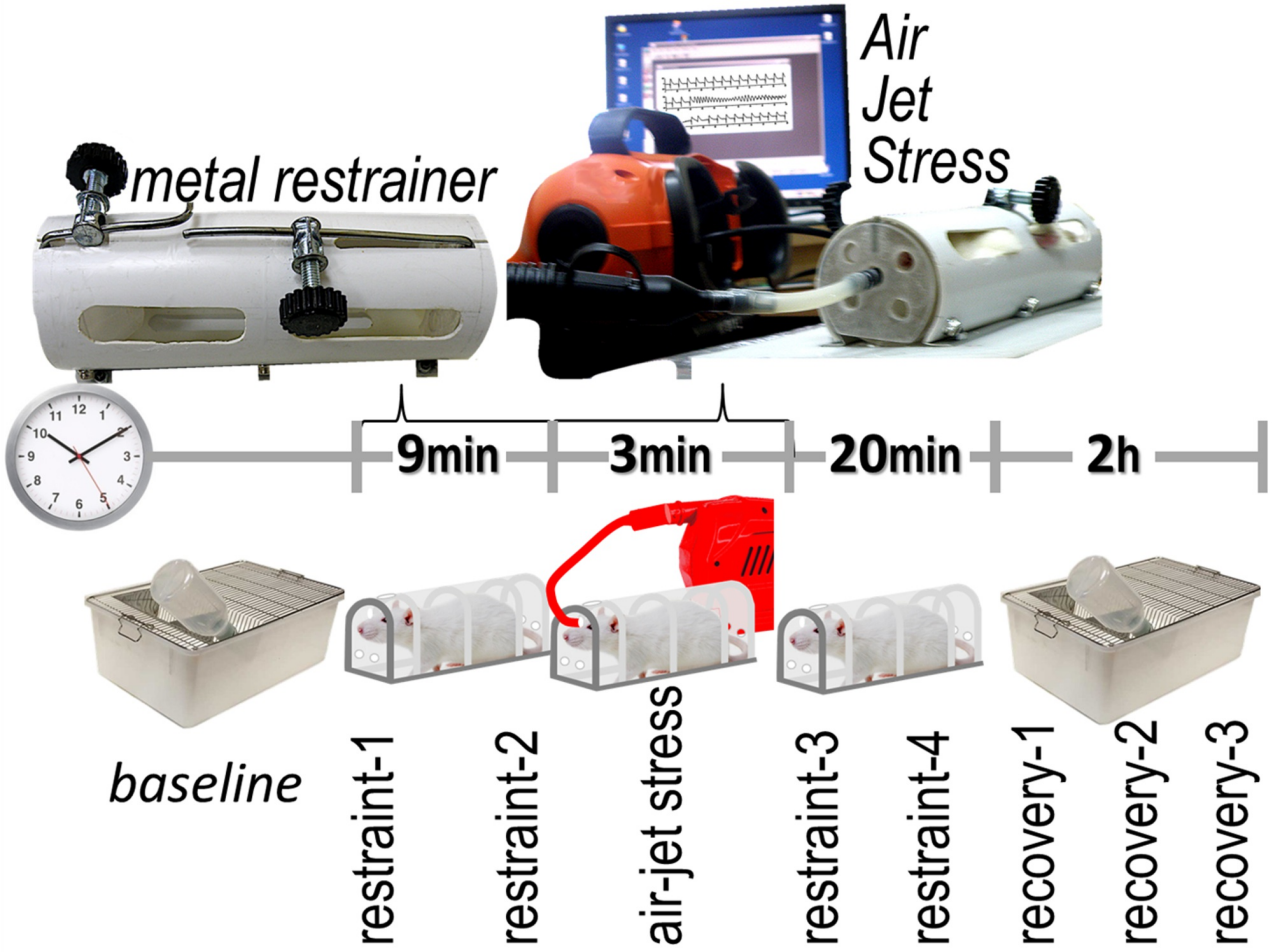
Protocol of the study The stress protocol included the successive use of restraint and air-jet stress. Autonomic variables were evaluated at nine time frames, each of three-minute duration.

One day prior to the induction of stress, the cages (containing rats with implanted transmitters) were placed on top of telemetry receivers (RCA-1020, *Transoma*), through which the ECG signal was recorded by the acquisition software (ART, *Transoma*). The room was kept quiet, maintaining the light/dark cycles, with all experiments performed during the morning hours. The stress protocol used in our experiments (Figure 1), adopted from previous descriptions, consisted of restraint [3] followed by air-jet stress [4], with a total duration of 32 minutes.

The rats were placed in a tubular restrainer (inner diameter: 8.25cm) with sufficient ventilation. Nine minutes thereafter, air-jet stress was initiated for three minutes, consisting of 18 pulses (each of two-second duration, delivered at eight-second intervals). Specifically, air was blown (at 10 L/min) to the forehead by a pump (BDINF20C, Black & Decker, Towson, MD, USA) with its outlet attached to a 3.2 mm opening at the front of the restrainer. Restraint was sustained for further 20 minutes, after which period the rats were returned to their cages, under continuous ECG monitoring for two hours. The following three-minute periods were analyzed: baseline, restraint-1, restraint-2, air-jet, restraint-3, restraint-4, recovery-1, recovery-2, and, recovery-3. In control experiments, the operator entered the room and observed the animals (from a distance of approximately one meter) for 32 minutes, with measurements corresponding to the time periods of the stress protocol.

Heart rate variability

HRV analysis was performed from consecutive inter-beat intervals, with the use of the *Kubios *software (University of Eastern Finland, Kuopio, Finland); indices at each period were averaged from three-minute recording intervals. As no single HRV index provides accurate description of the activation of each autonomic arm, we used those derived from a combination of variables, as previously outlined [8]. In brief, the sympathetic nervous system index (SNSi) was computed from three variables, namely (a) mean HR, (b) Baevsky’s stress index, and (c) the *length *of the distribution of Poincaré plots after nonlinear analysis. The parasympathetic nervous system index (PNSi) was computed also from three variables, namely (a) the mean inter-beat interval, (b) the root mean square of successive differences between inter-beat intervals in time-domain analysis, and (c) the *width *of the distribution of Poincaré plots. Individual variability in SNSi and PNSi responses was accounted for by their expression as percent change from baseline values.

Voluntary activity was recorded with the use of the analysis software (ART, *Transoma*) for the two-hour period post-stress. This variable provides a measure of continuing anxiety and is used as a marker of post-stress adaptation [6]. We report the total number of motion counts calculated by the analysis software, derived from strength variations in the telemetry signal in relation to animal location.

Statistical analysis

Values are reported as mean ± standard error of the mean. Baseline autonomic values and voluntary activity post-stress in the two groups were compared with t-test. The fluctuations of autonomic variables over time were assessed with the use of the (two-way) analysis of variance for repeated measures, with *stress *and *time *as between- and within-groups factors, respectively. The results are presented separately for each effect, along with their respective F-values (i.e., the ratio between the variation between sample means and variation within the samples) and p values. Differences (between- and within-groups) at each prespecified time period were evaluated with the use of post-hoc Duncan’s multistage test, with statistical significance set at an alpha level of 0.05.

## Results

The implantation of transmitters preceded the stress experiments by (a similar time period of) 6.0 ± 0.2 and 5.8 ± 0.3 days in the stress and control groups, respectively. At the end of this period, all animals showed signs of complete recovery.

Heart rate

**Figure 2:**
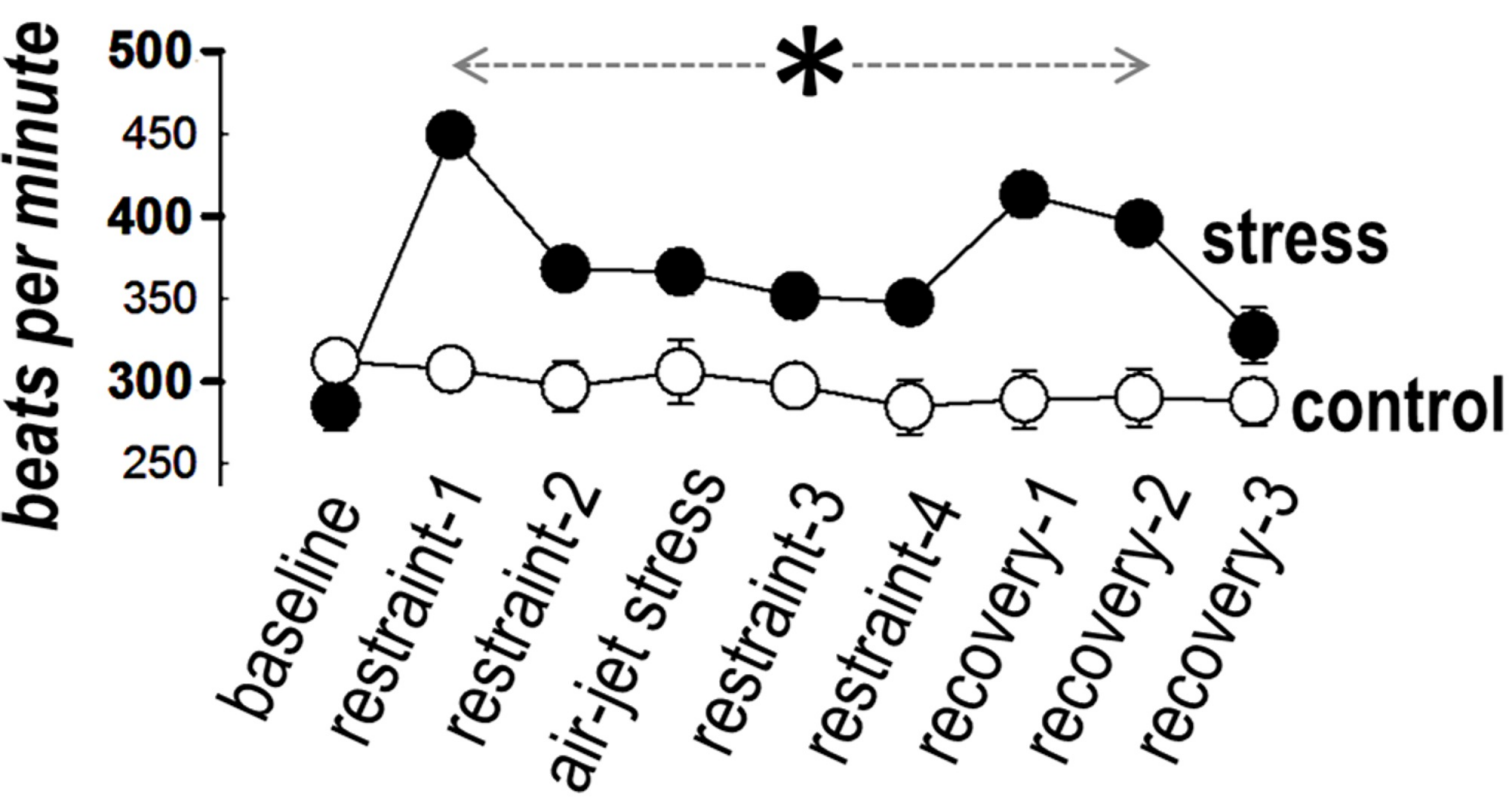
Heart rate Heart rate increased in the stress group immediately after restraint and remained higher than controls at all time frames (asterisk), except for the last three minutes of recovery.

HR was comparable between the two groups at baseline. As seen in Figure 2, HR remained stable in the control group throughout the observation period (F = 0.6, p = 0.7). By contrast, significance variance over time was present in the stress group (F = 36.1, p < 0.001). Specifically, HR increased in this group immediately after restraint (restraint-1) and remained elevated at all time frames, including the recovery period. Between-groups comparison revealed higher HR in the stress group than in controls throughout restraint and air-jet, with comparable values seen only during the last three minutes of recovery (Figure 2).

Sympathetic response

**Figure 3:**
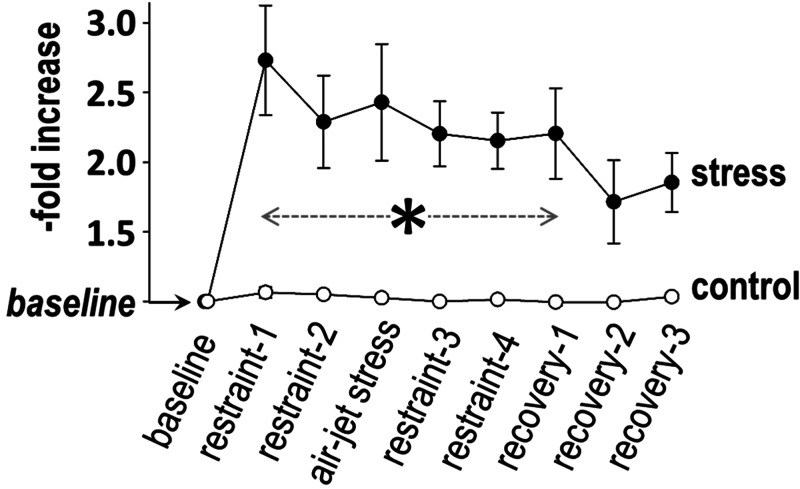
Sympathetic response The sympathetic nervous system index was higher in the stress group than in the controls (asterisk) during the entire period of stress and at the beginning of recovery.

SNSi was comparable between the two groups at baseline. The SNSi during stress and recovery (as percent change from baseline) is shown in Figure 3; SNSi remained stable in controls over time (F = 1.3, p = 0.2), but a significant variance was present in the stress group (F = 3.3, p = 0.005); of note, a wide range of responses was observed within this group. Between-group comparisons revealed higher SNSi in the stress group (than in controls) during the entire period of stress and at the beginning of recovery (Figure 3).

Vagal response

**Figure 4:**
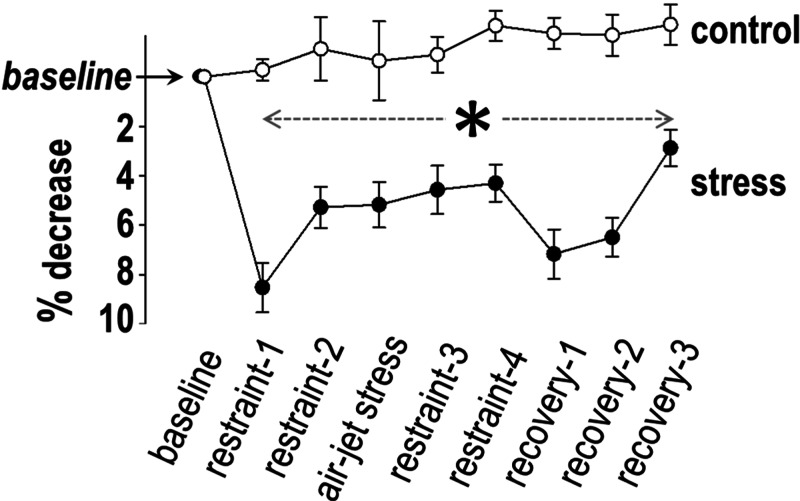
Vagal response The parasympathetic nervous system index was lower in the stress group than in the controls during stress *and *recovery (asterisk).

PNSi was comparable between the two groups at baseline. PNSi remained stable in controls over time (F = 0.7, p = 0.6), but a significant variance was present in the stress group (F = 20.6, p < 0.0001), characterized by low variation within the samples. The temporal pattern of PNSi in this group displayed a marked decrease after the onset of acute stress, followed by low values during the remaining period of observation (Figure 4). As a result, between-group comparisons revealed lower PNSi in the stress group (than in controls) throughout the period of acute stress and recovery.

Voluntary motion during recovery

Voluntary motion during the two-hour recovery period was more pronounced in the stress group than in the controls. Specifically, the total number of motion counts was higher (p = 0.035) in the former (8878 ± 1715) than in the latter group (2790 ± 1186).

## Discussion

Acute emotional stress evokes autonomic responses [9,10], but insufficient information is available on their precise nature and time course. In the present study, we analyzed sympathetic and vagal indices during and after acute unpredictable stress in rats.

Heart rate

We observed an initial rise in HR (by approximately 50%) in the stress group, with high values (at approximately 25% above baseline) persisting during the entire period of restraint and air-jet. These findings compare favorably with those previously observed in a similar protocol, consisting of 10 minutes of restraint followed by 10 minutes of air-jet stress [11]. Likewise, HR increased (by approximately 30%) during air-jet stress in another study [12], albeit no change was noted during an initial 10-minute restraint period. This slight diversity in HR responses may be attributed to minor methodological differences, such as the size and shape of the restrainer. Overall, the combination of restraint with air-jet in rats appears an effective model of acute stress, consistently increasing HR (as an index of sympathovagal balance) by approximately 30%. 

HRV analysis

The most important contribution of our study was the separate assessment of each autonomic arm. This was performed non-invasively in conscious animals, thereby overcoming the confounding effects of anesthesia. We opted to avoid the use of HRV analysis in the frequency domain (after fast Fourier transformation), based on previous reservations regarding its accuracy in separating sympathetic from vagal activation [13]. Instead, we used the more precise indices of sympathetic (SNSi) and vagal (PNSi) activation, calculated from multiple variables [8].

Sympathetic response

Following an initial pronounced rise, sympathetic activation reached a plateau during the successive use of restraint and air-jet in our experiments. Nonetheless, it should be noted that a wide range of individual responses was observed during stress, in keeping with previous descriptions in animal models [14] and humans [15]. This observation may have important implications in the pathophysiology of stress-induced arrhythmogenesis and calls for further investigation of the factors determining the magnitude of sympathetic activation in response to various stimuli [16].

Vagal response

Contrasting the SNSi pattern, PNSi responses in our stress group were less pronounced, but they displayed narrow inter-individual variability. Specifically, we observed a decrease by approximately 10% in PNSi at the onset of restraint, with low values persisting during the remaining period of stress. Interestingly, the transfer to the cage at the onset of recovery was accompanied by further decline in PNSi, reinforcing previous conclusions on swift vagal responses following stressful stimuli [17,18]. Such responses are important, given the potent vagal effects on ventricular electrophysiology, exerted via changes in action potential duration, refractory period, and fibrillation threshold [16].

Recovery from stress

The time period immediately following *physical *stress has been extensively studied, with HR recovery considered a robust prognostic marker in various cohorts. Similar to exercise, the importance of recovery after *emotional *stress has been also brought forward [19] and bears clinical relevance with the well-described delayed arrhythmogenesis post-stress [1,2]; hence, our protocol included a two-hour observation period, during which voluntary activity was also recorded, as an index of continuing anxiety [6].

We report two distinct patterns of recovery in the two autonomic arms. Specifically, SNSi declined gradually post-stress, although the wide variation in responses persisted; by contrast, vagal withdrawal was present during the entire post-stress period, evidenced by lower PNSi values than in controls. Such pattern indicates continuing anxiety with predominantly low parasympathetic activity, a conclusion supported by the markedly enhanced voluntary motion in the stress group.

Strengths and limitations

Our study presents three major strengths: *first*, we accounted for the previously reported gender-related differences in response to stress; the explanation for this observation is multifaceted, and likely includes the differential effects of sex hormones on nitric oxide handling [7]. *Second*, we used the noninvasive SNSi, which permits prompt identification of sympathetic changes without interfering with the stress protocol, thereby placing an advantage over plasma catecholamine measurements. *Third *and foremost, the PNSi, also calculated as part of the HRV analysis, provides information on vagal status, which constitutes an integral part of autonomic responses after emotional stress.

The main limitation of our protocol is the absence of concurrent assessment of the hypothalamic-pituitary-adrenal axis, a key element of stress response, mediated by the release of glucocorticoids from the adrenal cortex. Nonetheless, the translational relevance of our protocol was focused on the effects of stress on cardiac electrophysiology; in this regard, autonomic changes secondary to acute emotional stress have been implicated in two important arrhythmogenic mechanisms involving ventricular repolarization, namely its restitution properties at progressively shorter inter-beat intervals (i.e., at high HR) and its dispersion across the myocardium [20].

## Conclusions

We investigated the effects of acute stress on autonomic function in rats, using noninvasive indices derived from HRV analysis of continuous ECG recordings. We found prominent sympathetic activation during the entire period of stress, albeit considerable inter-individual variation was noted. Vagal withdrawal was consistent during this period. The recovery was slow over a two-hour observational period, characterized by continuing anxiety and low parasympathetic activity. Our findings emphasize the importance of detailed evaluation of autonomic responses, particularly in the investigation of arrhythmogenesis induced by aversive stimuli.
